# Complete Genome Sequences of Two Methicillin-Susceptible Staphylococcus aureus Clinical Strains Closely Related to Community-Associated Methicillin-Resistant S. aureus USA300

**DOI:** 10.1128/MRA.00356-19

**Published:** 2019-04-25

**Authors:** Jo-Ann McClure, Kunyan Zhang

**Affiliations:** a Centre for Antimicrobial Resistance, Alberta Health Services/Alberta Public Laboratories/University of Calgary, Calgary, Alberta, Canada; b Department of Pathology & Laboratory Medicine, University of Calgary, Calgary, Alberta, Canada; c Department of Microbiology, Immunology and Infectious Diseases, University of Calgary, Calgary, Alberta, Canada; d Department of Medicine, University of Calgary, Calgary, Alberta, Canada; e The Calvin, Phoebe and Joan Snyder Institute for Chronic Diseases, University of Calgary, Calgary, Alberta, Canada; University of Rochester School of Medicine and Dentistry

## Abstract

Predominant community-associated methicillin-resistant Staphylococcus aureus strain USA300 is believed to have originated from an ancestral methicillin-susceptible strain, although the details of that evolution remain unknown. To help understand the emergence of this highly successful strain, we sequenced the genomes of two methicillin-susceptible Staphylococcus aureus clinical strains that are very closely related to USA300.

## ANNOUNCEMENT

The evolutionary origins of the major methicillin-resistant Staphylococcus aureus (MRSA) clones are still poorly understood, although it is hypothesized that they repeatedly arose from epidemic methicillin-susceptible S. aureus (MSSA) strains through acquisition of the *mecA* gene via horizontal transfer (1–5). The highly successful community-associated MRSA (CA-MRSA) strain USA300 has become predominant in North America, causing significant morbidity and mortality (6–12). It is believed to have descended from an ancestral USA500-like MSSA strain through acquisition of multiple mobile genetic elements (MGEs) and clonal expansion (13, 14). As with the other major MRSA clones, more work is needed to fully understand its emergence and success. To that end, we selected two MSSA isolates that are closely related to USA300 for whole-genome sequencing, with the goal of elucidating the genetic and evolutionary relationships between these MSSA isolates and the highly successful USA300 MRSA group. Strain H489 was isolated by our clinical microbiological laboratory from the sputum of a patient from our local health care region in Calgary, Canada, in 1993, well before our USA300 outbreak began in 2004. Likewise, strain C3948 was isolated from a patient in 2002, just before the USA300 outbreak. Multilocus sequence analysis of the isolates indicated that, similarly to the USA300 outbreak strain, they belonged to sequence type 8 (ST8).

Genomic DNA was isolated by phenol-chloroform extraction of overnight cultures started from a single colony. Library preparation, DNA sequencing, contig assembly, and genome circularization were performed at the Génome Québec Innovation Centre in Montreal, Canada. Sheared large-insert libraries were prepared with Covaris g-TUBES and the SMRTbell template prep kit 1.0. Sequencing was done using the Pacific Biosciences (PacBio) RSII sequencing technology, with one single-molecule real-time (SMRT) cell. Contig assembly was done using the RS Hierarchical Genome Assembly Process (HGAP) protocol version 2.3.0.140936.p5 (15–17), with read quality controlled by aligning short reads on longer reads using BLASR (15). The genomes were circularized using Circlator version 1.4.1 and adjusted to the origin of replication (18). Gene annotation was done using the NCBI’s Prokaryotic Genome Annotation Pipeline version 4.1, using the best-placed reference protein set (GeneMarkS+) (19).

Two contigs were assembled for MSSA strain C3948 from 85,037 raw reads, covering 1,142,835,600 sequenced bases, with an *N*_50_ value of 2,823,074 bp. The estimated genome coverage was 368×, and the GC content was 32.82%. On the assembled chromosome of 2,795,888 bp, 2,924 genes were identified, of which 2,842 were coding sequences (CDS), 82 were RNA genes, and 76 were pseudogenes. Four contigs were assembled for MSSA strain H489 from 94,559 raw reads covering 1,215,818,661 sequenced bases, with an *N*_50_ value of 2,761,569 bp. The estimated genome coverage was 392×, and the GC content was 32.79%. On the chromosome of 2,757,748 bp, 2,874 genes were identified, of which 2,789 were CDS, 85 were RNA genes, and 94 were pseudogenes.

A complete analysis is under way to look at the major genetic components in these MSSA isolates and compare them with those found in MRSA USA300 to help shed light on the evolutionary path of the highly successful USA300 strain.

### Data availability.

The chromosomal genome sequences have been deposited at GenBank under the accession numbers CP020957 (C3948) and CP020959 (H489), with SRA accession numbers SRX5551895 (C3948) and SRX5552203 (H489).
